# Influence of Graft Type on Muscle Contractile Dynamics After ACL Reconstruction: A 9-Month Tensiomyographic Follow-Up

**DOI:** 10.3390/diagnostics15151920

**Published:** 2025-07-30

**Authors:** Georges Kakavas, Florian Forelli, Yoann Demangeot, Vasileios Korakakis, Nikolaos Malliaropoulos, Nicola Maffulli

**Affiliations:** 1 Fysiotek, Spine and Sports Lab, 11635 Athens, Greece; georgios.kakavas@gmail.com; 2 Haute-Ecole Arc Santé, HES-SO University of Applied Sciences and Arts Western Switzerland, 2000 Neuchâtel, Switzerland; 3 Orthopaedic Surgery Department, Clinic of Domont, Ramsay Healthcare, OrthoLab, 95330 Domont, France; 4 SFMK Lab, 95270 Asnières sur Oise, France; 5 University of Reims Champagne-Ardenne, MATIM, 51100 Reims, France; ydemangeot@chu-reims.fr; 6 Faculty of Biology and Medicine, University of Lausanne, 1015 Lausanne, Switzerland; 7 Department of Physical and Rehabilitation Medicine, Reims University Hospital, Sébastopol Hospital, 51100 Reims, France; 8 Department of Health Sciences, School of Life Sciences and Health Sciences, PhD in Physiotherapy Program, University of Nicosia, Nicosia 2417, Cyprus; vkorakakis@hotmail.com; 9 Center of Orthopaedics and Regenerative Medicine (C.O.RE.)/(C.I.R.I.)—Aristotle University Thessaloniki, 10431 Hellas, Greece; contact@sportsmed.gr; 10 Centre for Sports and Exercise Medicine, Queen Mary University of London, London E1 4NS, UK; n.maffulli@qmul.ac.uk; 11 Sports Clinic, Mile End Hospital, Barts and the London, London E1 4DG, UK; 12 Department of Trauma and Orthopaedic Surgery, Faculty of Medicine and Psychology, Sapienza University, 00185 Roma, Italy; 13 Guy Hilton Research Centre, Institute of Science and Technology in Medicine, School of Medicine, Keele University, Thornburrow Drive, Hartshill, Stoke-on-Trent ST4 7QB, UK

**Keywords:** anterior cruciate ligament reconstruction, tensiomyography, graft type, muscle recovery, neuromuscular assessment

## Abstract

**Background**: Persistent neuromuscular deficits following anterior cruciate ligament reconstruction (ACLR) are frequently attributed to arthrogenic muscle inhibition (AMI). The type of autologous graft used may influence the trajectory of neuromuscular recovery. **Objective**: To investigate the influence of graft type—bone–patellar tendon–bone (BPTB), hamstring tendon (HT), and quadriceps tendon (QT)—on the contractile properties of periarticular knee muscles over a 9-month post-operative period. **Hypothesis**: Each graft type would result in distinct recovery patterns of muscle contractility, as measured by tensiomyography (TMG). **Methods**: Thirty-one patients undergoing ACLR with BPTB (*n* = 8), HT (*n* = 12), or QT (*n* = 11) autografts were evaluated at 3, 6, and 9 months post-operatively. TMG was used to measure contraction time (Tc) and maximal displacement (Dm) in the rectus femoris, vastus medialis, vastus lateralis, and biceps femoris. **Results**: Significant within-group improvements in Tc and Dm were observed across all graft types from 3 to 9 months (Tc: *p* < 0.001 to *p* = 0.02; Dm: *p* < 0.001 to *p* = 0.01). The QT group showed the most pronounced Tc reduction in RF (from 30.16 ± 2.4 ms to 15.44 ± 1.6 ms, *p* < 0.001) and VM (from 31.05 ± 2.6 ms to 18.65 ± 1.8 ms, *p* = 0.004). In contrast, HT grafts demonstrated limited Tc recovery in BF between 6 and 9 months compared to BPTB and QT (*p* < 0.001), indicating a stagnation phase. BPTB exhibited persistent bilateral deficits in both quadriceps and BF at 9 months. **Conclusions**: Autograft type significantly influences neuromuscular recovery patterns after ACLR. TMG enables objective, muscle-specific monitoring of contractile dynamics and may support future individualized rehabilitation strategies.

## 1. Introduction

Anterior cruciate ligament (ACL) injuries represent one of the most prevalent and impactful musculoskeletal injuries encountered in sports medicine [1]. These injuries are particularly common in high-demand activities that involve cutting, pivoting, deceleration, and jumping, such as soccer, basketball, and alpine skiing [2,3]. The rate of ACL reconstructions (ACLR) has steadily risen over the past two decades, reflecting both increased injury incidence and the widespread adoption of surgical repair as a standard of care, especially among younger and more active individuals [4]. While ACLR typically restores passive joint stability and prevents further intra-articular damage, a large number of patients report lingering neuromuscular impairments and difficulties returning to pre-injury performance levels [5,6]. A key factor contributing to these deficits is arthrogenic muscle inhibition (AMI), a protective reflex mechanism in which the excitability of alpha motor neurons is reduced due to altered sensory input following joint trauma [7]. AMI disrupts voluntary muscle activation—most notably in the quadriceps—even when muscle tissue remains structurally intact [8]. As a result, it leads to persistent strength deficits, impaired neuromuscular control, and higher re-injury rates [9,10]. The clinical consequences of AMI are significant: it slows rehabilitation progress, reduces physical function, alters movement patterns, and potentially contributes to early onset of osteoarthritis [11,12,13]. The precise degree of AMI can vary significantly between individuals, suggesting that surgical and rehabilitation variables—such as graft type—may modulate the severity and duration of this neuromuscular dysfunction [14,15].

Autologous graft selection is a central decision in ACLR and may directly influence the development of AMI. The three most common grafts used are the bone–patellar tendon–bone (BPTB), hamstring tendon (HT), and quadriceps tendon (QT) [16]. Each of these grafts has unique structural characteristics and implications for donor-site morbidity, healing profiles, and regional muscle trauma. BPTB grafts provide excellent initial fixation strength via bone-to-bone healing but are frequently associated with anterior knee pain, kneeling discomfort, and quadriceps inhibition, potentially exacerbating AMI [17]. HT grafts, which involve harvesting the semitendinosus and gracilis tendons, generally reduce anterior knee symptoms but may impair knee flexor strength and rotational stability [18]. QT grafts are gaining traction as a middle-ground option due to their large cross-sectional area and acceptable morbidity, but data on their neuromuscular effects, particularly on AMI, remain limited. Several studies have assessed muscle strength recovery after ACLR in relation to graft type, but the vast majority rely on isokinetic dynamometry or surface electromyography [19,20,21,22,23]. While these tools provide important functional insights, they may not detect more subtle impairments in reflexive muscle control [6]. Moreover, the existing literature tends to focus on isolated quadriceps performance, neglecting a broader assessment of periarticular muscle groups, or lacks a longitudinal framework. Very few studies explore how AMI evolves over time across different graft types using objective, muscle-specific diagnostic tools [15,24,25]. Tensiomyography (TMG), a non-invasive technique for assessing muscle contractile properties such as contraction time (Tc) and maximal displacement (Dm), offers a promising method to fill this gap [19,20]. TMG can detect changes in muscle stiffness, neuromuscular responsiveness, and fiber-type behavior, providing a deeper comprehension of functional muscle status during rehabilitation [26]. However, its use remains rare in clinical ACLR studies, and its potential to track AMI progression and recovery in relation to graft type is underexplored [6].

The objective of this study was to assess the effect of graft type (BPTB, HT, QT) on neuromuscular recovery following ACLR. TMG was used over a 9-month follow-up to evaluate changes in muscle contractility in key periarticular muscles (RF, VM, VL, BF). The aim was to identify graft-specific recovery patterns that could inform surgical choice and guide individualized rehabilitation approaches.

## 2. Methods

### 2.1. Study Design

This study was designed as a longitudinal observational cohort to evaluate neuromuscular recovery patterns following ACLR using different autologous graft types. Participants were assessed at three time points—3, 6, and 9 months post-operatively—using TMG to measure contractile properties of periarticular knee muscles. The study was approved by the Institutional Review Board of the University of Thessaly (Approval Number: #10234) and all procedures were conducted in accordance with the Declaration of Helsinki. Written informed consent was obtained from all participants prior to inclusion.

### 2.2. Participants

Thirty participants (15 males, 16 females; mean age: 21.74 ± 1.67 years) who had undergone primary ACLR were enrolled. Inclusion criteria were as follows: (1) unilateral, first-time ACL rupture confirmed clinically and radiologically; (2) ACLR using an autologous BPTB, HT, or QT graft; (3) age 18–30 years; and (4) availability for follow-up assessments. Exclusion criteria included bilateral ACL injuries, previous knee surgery, or use of allograft/synthetic grafts. The age range of 18–30 years was chosen to ensure a relatively homogeneous cohort in terms of neuromuscular function, physical activity level, and recovery potential. This age group also represents the most frequent candidates for ACLR in sports medicine, allowing for a more consistent analysis of graft-specific recovery patterns.

Sample size was estimated to be a priori using G*Power v3.1.9.7. A Kruskal–Wallis H test was selected for inter-group comparisons with three groups, assuming a moderate-to-large effect size (f = 0.4), α = 0.05, and power = 0.80. The required sample size was 66 participants (22 per group). Due to recruitment constraints, 30 participants were enrolled (BPTB: 8; HT: 12; QT: 11), which limits subgroup statistical power. This study is thus exploratory and intended to inform future larger-scale research.

Although all patients underwent post-operative rehabilitation, the specific content, frequency, and progression of rehabilitation protocols were not standardized nor recorded as part of the study.

### 2.3. Graft Types

Graft selection was determined based on shared decision-making between the patient and surgical team, considering sport level, anatomy, previous injuries, and surgeon preference. All surgeries were performed arthroscopically using standardized techniques by experienced orthopedic surgeons. Participants were stratified into three graft groups: BPTB (*n* = 8), HT (*n* = 12), and QT (*n* = 11).

### 2.4. Instrumentation and Measurements

TMG (TMG Performance System, Ljubljana, Slovenia) was used to assess muscle contractile characteristics in the rectus femoris, vastus medialis, vastus lateralis, and biceps femoris of the operated limb. TMG evaluates muscle response to a controlled electrical stimulus, providing key indicators: Tc (ms), reflecting contraction speed and fiber composition, and Dm (mm), reflecting muscle stiffness and displacement capacity.

### 2.5. Procedures

Participants were positioned supine or prone depending on the muscle tested. Electrodes and a digital displacement sensor were placed on the muscle belly after skin preparation. A single supramaximal electrical stimulus was applied [27,28,29]. Each muscle was measured three times per session, and the mean of the three values was used. All measurements were conducted by the same operator under standardized conditions.

### 2.6. Outcome Measures

Primary outcomes were Tc and Dm values for each of the four muscles, recorded at 3, 6, and 9 months post-ACLR. The percentage change in Tc and Dm between 3 and 9 months was calculated to assess recovery trends and AMI resolution. Inter-group comparisons (BPTB vs. HT vs. QT) were performed at each time point, along with intra-group longitudinal analyses.

### 2.7. Statistical Analysis

All analyses were conducted using JASP (version 0.19.3, Amsterdam, The Netherlands). Descriptive statistics (mean, SD) were calculated for all variables. Normality was assessed using Shapiro–Wilk test. Since most variables were not normally distributed and the group sample sizes were small, non-parametric methods were used. For each group, a Friedman test for repeated measures was used to investigate the change over time of each variable (TC and DM). Kendall’s W were used to estimate effect size (0.1: small effect; 0.3: moderate effect; 0.5 and more: strong effect). Conover’s post hoc tests with Bonferroni correction were used if the Friedman test was significant. The Kruskal–Wallis H test was employed to detect differences across the three graft groups at each test period (3–6–9 months). Where significant, Dunn’s post hoc comparisons were made with Bonferroni correction. Significance was set at *p* < 0.05.

## 3. Results

### 3.1. Participant Characteristics

Baseline characteristics of the study population are presented in Table 1. The mean age of the patients was 21.74 ± 1.67 years, and no significant differences were found for the variables of age (*p* = 0.41), height (*p* = 0.14), weight (*p* = 0.10), and BMI (*p* = 0.14).

### 3.2. Contraction Time (Tc)

**Figure 1:**
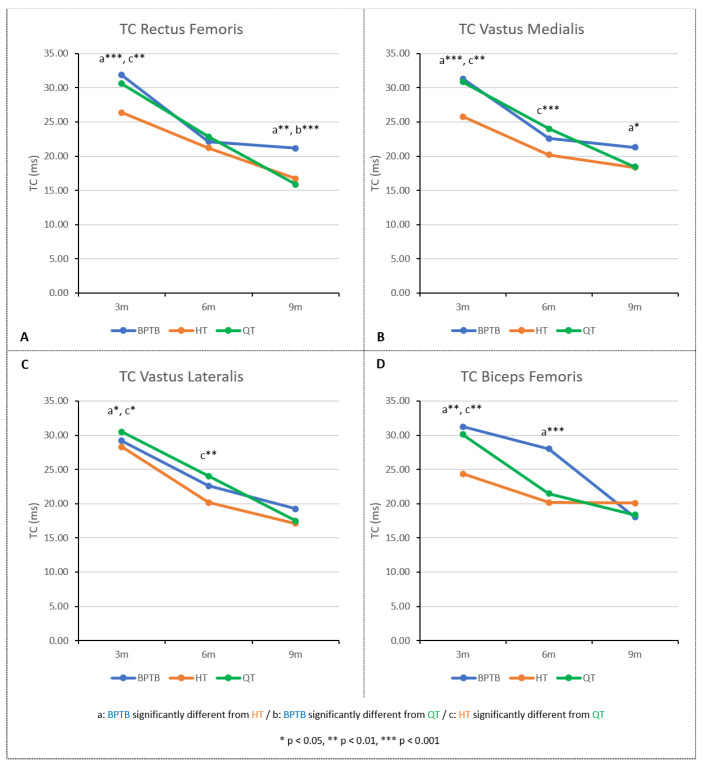
TC values for rectus femoris (**A**), vastus medialis (**B**), vastus lateralis (**C**), and biceps femoris (**D**). BPTB, bone–patellar tendon–bone; HT, hamstrings tendon; QT, quadriceps tendon.

- *Rectus femoris (Figure 1A*)

There was a significant time effect on rectus femoris TC values (BPTB: χ^2^ (2) = 13.00, *p* = 0.002, Kendall’s W = 0.713; HT: χ^2^ (2) = 24.00, *p* < 0.001, Kendall’s W = 1.00; QT: χ^2^ (2) = 22.00, *p* < 0.001, Kendall’s W = 1.00). For each graft, the Conover’s post hoc tests showed a significant reduction in TC values between all test periods (*p* < 0.001), except for the 3 to 6 months period for BPTB graft (*p* = 0.146) (Table 2).

At 3 and 9 months, the Kruskal–Wallis H test indicated that there are significant differences between the rectus femoris TC values among the three grafts groups (3 months: *H*(2) = 21.10, *p* < 0.001; 9 months: *H*(2) = 18.63, *p* < 0.001). The pairwise post hoc Dunn test with Bonferroni adjustments showed that (1) at 3 months the HT group had significantly lower TC values than the BPTB and QT groups (*p* < 0.001 and *p* < 0.01, respectively), and (2) at 9 months the BPTB group had significantly higher values than the HT and QT groups (*p* < 0.01 and *p* < 0.001, respectively). Other pairwise comparisons showed no significant differences, and no significant differences were found between the TC values among the three grafts groups at 6 months (*H*(2) = 5.93, *p* = 0.05).

- *Vastus Medialis (Figure 1B)*

There was a significant time effect on vastus medialis TC values (BPTB: χ^2^ (2) = 12.250, *p* = 0.002, Kendall’s W = 0.766; HT: χ^2^ (2) = 16.167, *p* < 0.001, Kendall’s W = 0.674; QT: χ^2^ (2) = 18.727, *p* < 0.001, Kendall’s W = 0.851). For each graft, the Conover’s post hoc tests showed a significant reduction in TC values between all test periods, except for the 6 to 9 months period for BPTB and HT grafts (*p* = 1.00 and *p* = 0.304, respectively) (Table 2).

TC values were significantly different among the three graft groups at 3, 6 and 9 months (3 months: *H*(2) = 22.54, *p* < 0.001; 6 months: *H*(2) = 13.84, *p* < 0.001; 9 months: *H*(2) = 8.14, *p* < 0.05). Dunn’s post hoc comparisons showed the following: (1) at 3 months, the HT group had significantly lower TC values than the BPTB and QT groups (*p* < 0.001 and *p* < 0.01, respectively); (2) at 6 months, the HT group had significantly lower TC values than the QT group (*p* < 0.001); and (3) at 9 months, the BPTB group had significantly higher TC values than the HT group (*p* < 0.05). Other pairwise comparisons showed no significant differences (Table 2).

- *Vastus Lateralis (Figure 1C)*

There was a significant time effect on vastus lateralis TC values (BPTB: χ^2^ (2) = 13.267, *p* = 0.001, Kendall’s W = 0.829; HT: χ^2^ (2) = 18.681, *p* < 0.001, Kendall’s W = 0.778; QT: χ^2^ (2) = 22.000, *p* < 0.001, Kendall’s W = 1.000). For each graft, the Conover’s post hoc tests showed a significant reduction in TC values between all test periods (Table 2).

The vastus lateralis TC values were significantly different among the three graft groups at 3 and 6 months (*H*(2) = 9.05, *p* < 0.05, and *H*(2) = 9.57, *p* < 0.01, respectively), but not at 9 months (*H*(2) = 3.67, *p* = 0.16). Dunn’s post hoc comparisons showed that the HT group had significantly lower TC values than the QT group at 3 and 6 months (*p* < 0.05 and *p* < 0.01, respectively) and had significantly lower TC values than the BPTB group at 3 months only (*p* < 0.05). Other pairwise comparisons showed no significant differences (Table 2).

- *Biceps Femoris (Figure 1D)*

There was a significant time effect on biceps femoris TC values (BPTB: χ^2^ (2) = 10.516, *p* = 0.005, Kendall’s W = 0.657; HT: χ^2^ (2) = 12.667, *p* = 0.002, Kendall’s W = 0.528; QT: χ^2^ (2) = 17.636, *p* < 0.001, Kendall’s W = 0.802). The Conover’s post hoc tests showed significant reduction in TC values between 3 and 9 months for all groups. TC values significantly decreased between 3 and 6 months for both HT and QT groups (*p* < 0.001), but not for the BPTB group (*p* = 0.380). Conversely, only the BTPB group’s TC values decreased significantly between 6 and 9 months (*p* = 0.012) (Table 2).

The biceps femoris TC values were significantly different among the three graft groups at 3 and 6 months (*H*(2) = 15.03, *p* < 0.001, and *H*(2) = 13.89, *p* < 0.001, respectively), but not at 9 months (*H*(2) = 1.07, *p* = 0.59). Dunn’s post hoc comparisons showed that the HT group had significantly lower TC values than the BPTB group at 3 and 6 months (*p* < 0.01 and *p* < 0.001, respectively) and had significantly lower TC values than the QT group at 3 months (*p* < 0.01). Other pairwise comparisons showed no significant differences (Table 2).

### 3.3. Maximal Displacement (Dm)

**Figure 2:**
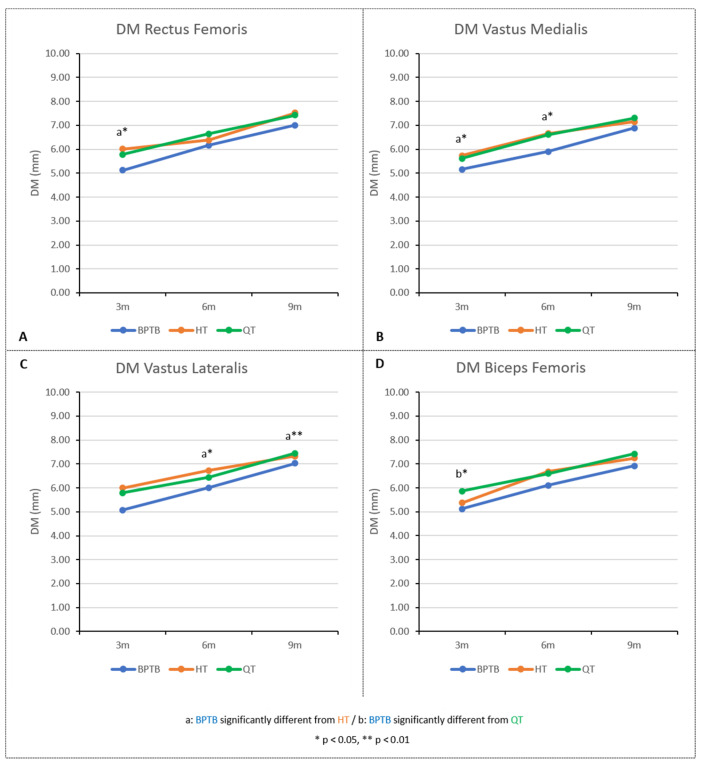
DM values for rectus femoris (**A**), vastus medialis (**B**), vastus lateralis (**C**), and biceps femoris (**D**). BPTB, bone–patellar tendon–bone; HT, hamstrings tendon; QT, quadriceps tendon.

- *Rectus femoris (Figure 2A)*

There was a significant time effect on rectus femoris DM values (BPTB: χ^2^ (2) = 10.750, *p* = 0.005, Kendall’s W = 0.672; HT: χ^2^ (2) = 17.783, *p* < 0.001, Kendall’s W = 0.741; QT: χ^2^ (2) = 11.636, *p* = 0.003, Kendall’s W = 0.529). The Conover’s post hoc tests showed no difference in DM values between 3 and 6 months, whatever the graft considered. Between 6 and 9 months, rectus femoris DM values increased significantly for BPTB and HT groups (*p* = 0.017 and *p* < 0.001, respectively), but no difference was observed for the QT group (*p* = 0.084). However, between 3 and 9 months, the rectus femoris DM values increased significantly, for the three graft groups (*p* < 0.001) (Table 2).

The rectus femoris DM values were significantly different among the three graft groups at 3 months (*H*(2) = 6.83, *p* < 0.05), but not at 6 and 9 months (6 months: *H*(2) = 5.37, *p* = 0.07; 9 months: *H*(2) = 5.46, *p* = 0.07). Dunn’s post hoc comparisons showed that the BPTB group had significantly lower DM values than the HT group at 3 months (*p* < 0.05). No significant difference was found between the BPTB and QT groups (*p* = 0.44), nor between the HT and QT groups (*p* = 0.65).

- *Vastus Medialis (Figure 2B)*

There was a significant time effect on vastus medialis DM values (BPTB: χ^2^ (2) = 12.250, *p* = 0.002, Kendall’s W = 0.766; HT: χ^2^ (2) = 18.558, *p* < 0.001, Kendall’s W = 0.773; QT: χ^2^ (2) = 12.698, *p* = 0.002, Kendall’s W = 0.577). For each graft, the Conover’s post hoc tests showed significant increase in DM values between all test periods, except for the 6 to 9 months period for QT graft (*p* = 0.084) (Table 2).

The vastus medialis DM values were significantly different among the three graft groups at 3 and 6 months (*H*(2) = 7.36, *p* < 0.05, and *H*(2) = 8.04, *p* < 0.05, respectively), but not at 9 months (*H*(2) = 1.60, *p* = 0.45). At 3 and 6 months, Dunn’s post hoc comparisons showed that the BPTB group had significantly lower DM values than the HT group (*p* < 0.05). Other pairwise comparisons showed no significant differences (Table 2).

- *Vastus Lateralis (Figure 2C)*

There was a significant time effect on vastus lateralis DM values (BPTB: χ^2^ (2) = 14.250, *p* < 0.001, Kendall’s W = 0.891; HT: χ^2^ (2) = 10.711, *p* = 0.005, Kendall’s W = 0.446; QT: χ^2^ (2) = 14.195, *p* < 0.001, Kendall’s W = 0.645). Between 3 and 6 months, the Conover’s post hoc tests showed increase in vastus lateralis DM values only for the BPTP group (*p* = 0.002). Between 6 and 9 months, rectus femoris DM values increased significantly for BPTB and QT groups (*p* < 0.001 and *p* = 0.001, respectively) but no difference was observed for the HT group (*p* = 0.212). Finally, between 3 and 9 months, the vastus lateralis DM values increased significantly for the three graft groups (*p* ≤ 0.001) (Table 2).

Vastus lateralis DM values were significantly different among the three graft groups at 6 and 9 months (*H*(2) = 6.76, *p* < 0.05, and *H*(2) = 9.88, *p* < 0.01, respectively), but not at 3 months (*H*(2) = 5.16, *p* = 0.08). At 6 and 9 months, Dunn’s post hoc comparisons showed that the BPTB group had significantly lower DM values than the HT group (*p* < 0.05 and *p* < 0.01, respectively). Other pairwise comparisons showed no significant differences (Table 2).

- *Biceps Femoris (Figure 2D)*

There was a significant time effect on biceps femoris DM values (BPTB: χ^2^ (2) = 12.250, *p* = 0.002, Kendall’s W = 0.766; HT: χ^2^ (2) = 19.478, *p* < 0.001, Kendall’s W = 0.812; QT: χ^2^ (2) = 12.333, *p* = 0.002, Kendall’s W = 0.561). For each graft, the Conover’s post hoc tests showed significant increase in biceps femoris DM values between all test periods, except for the 3 to 6 months period for QT graft (*p* = 0.164) and for the 6 to 9 months period for the HT graft (*p* = 0.238) (Table 2).

The biceps femoris DM values were significantly different among the three graft groups at 3 months (*H*(2) = 6.92, *p* < 0.05), but not at 6 and 9 months (6 months: *H*(2) = 3.52, *p* = 0.17; 9 months: *H*(2) = 5.99, *p* = 0.05). At 3 months, Dunn’s post hoc comparisons showed that the BPTB group had significantly lower DM values than the QT group (*p* < 0.05). No significant difference was found between the BPTB and HT groups (*p* = 0.23), nor between the HT and QT groups (*p* = 0.99).

## 4. Discussion

This study aimed to determine whether graft type (BPTB, HT, or QT) influences the evolution of muscle contractile properties after ACLR. The findings confirmed that all graft types demonstrated significant improvements in Tc and Dm between 3 and 9 months. However, the magnitude, timing, and muscle-specific patterns of recovery differed significantly between groups, suggesting that graft type modulates neuromuscular recovery profiles after ACLR.

Across all graft types, Tc decreased and Dm increased significantly in RF, VM, VL, and BF from 3 to 9 months. These changes reflect enhanced muscle responsiveness and reduced stiffness, consistent with the resolution of AMI and progressive reconditioning through rehabilitation [11,12,30,31,32,33,34]. Tc improvements were more pronounced between 3 and 6 months, with smaller but continued gains between 6 and 9 months. This biphasic recovery pattern aligns with prior descriptions of early neural reactivation followed by later structural remodeling [11,35,36].

At 3 months, Tc impairments were clearly graft-specific. Tc values were significantly higher in the BPTB and QT groups than in the HT group across all muscles, suggesting greater early corticospinal inhibition when grafts were harvested from the quadriceps tendon [17,18,20,37]. This inhibition is likely due to direct trauma from central tendon harvesting, which affects motor unit recruitment and excitability [17,20].

Interestingly, this inhibitory pattern extended beyond the quadriceps. BF also showed increased Tc in the BPTB and QT groups, despite not being directly involved in anterior graft harvesting. This is counterintuitive, as one might expect greater BF inhibition in the HT group, where the semitendinosus and gracilis—muscles functionally and neurologically related to BF—are harvested. However, in the HT group, BF had the lowest Tc (24.34 ± 3.03 ms), suggesting relatively preserved excitability in the early post-operative phase [18,37,38]. However, this initially favorable state in the HT group did not improve over time: BF showed no significant Tc change between 6 and 9 months (*p* = 1.000) [7], indicating stagnation in neuromuscular recovery rather than secondary inhibition.

The BPTB group, by contrast, exhibited a more diffuse inhibition pattern, with elevated Tc not only in quadriceps muscles but also in BF (31.25 ± 0.73 ms) at 3 months. This suggests that patellar tendon harvesting may disrupt both quadriceps activation and posterior chain compensation, possibly through broader neuromuscular or central mechanisms [17,39,40].

By 9 months, some differences had been resolved, while others persisted or reversed. In RF, VM, and VL, the QT group showed the greatest Tc reduction (∆Tc = −14.72 ± 1.53 ms, −12.39 ± 3.02 ms, and −13.00 ± 1.79 ms, respectively), suggesting a strong recovery trajectory [6,7]. In BF, inter-group differences in Tc were no longer significant at 9 months (*p* = 0.586), but the HT group displayed a plateau in recovery between 6 and 9 months, possibly indicating persistent inhibition [18,31,38]. Finally, it is worth noting that there is no longer any difference in Tc values between the HT and QT groups for all muscle groups at 9 months post-op, while the RF and VM Tc values of the BPTB group remain deficient.

The inter-group differences are less obvious in Dm. At 3 months, lower values were observed for the BPTB group compared to HT for RF and VM (*p* < 0.05), and compared to QT for BF muscle (*p* < 0.05). However, these differences were no longer persistent at 9 months. Conversely, VL Dm values were similar for all groups at 3 months, but lower for the BPTB group than for the QT group at 6 and 9 months (*p* < 0.05 and *p* < 0.01, respectively). By 9 months, most inter-group differences had diminished, reflecting progressive neuromuscular rebalancing. However, delayed recovery persisted in BF after HT grafting, and in RF, VM (Tc), and VL (Dm) after BPTB, indicating graft-specific residual deficits. Overall, however, there is a tendency for Dm values in the BPTB group to be lower than those in the other two groups, regardless of muscle group or test period.

The speed of neuromuscular recovery varied substantially by graft type. Although the QT group showed the highest Tc in VL at 3 months, it also demonstrated the steepest decline in VL Tc between 3 and 6 months (from 30.59 ± 1.33 ms to 22.83 ± 1.43 ms, *p* < 0.001), with continued improvement by 9 months (15.86 ± 0.70 ms). This profile suggests early inhibition followed by strong neural and muscular adaptation, confirming findings from previous TMG studies on QT grafts [7,20]. The HT group had a flatter Tc trajectory in BF, with a significant drop between 3 and 6 months (*p* < 0.001), but no further change from 6 to 9 months (*p* = 1.000). This delayed or incomplete recovery is consistent with the literature showing persistent hamstring deficits after semitendinosus–gracilis harvesting [18,32,38,41,42,43,44]. BPTB showed a more moderate Tc recovery of quadriceps muscles. In fact, RF and VM Tc deficits were still present at 9 months. VL Tc values also tended to be higher in the BPTB group than in the other two groups at 9 months, although this difference was not significant. In BF, Tc dropped slightly between 3 and 6 months (*p* = 0.380), followed by a sharper decline between 6 and 9 months (*p* = 0.012), suggesting delayed neural reactivation in the posterior chain, despite the graft not involving hamstring harvesting [6,7]. Dm followed a similar recovery pattern, though generally with slower improvements compared to Tc. In VL, for example, Dm increased significantly in both BPTB and QT groups from 3 to 9 months, while it plateaued in the HT group after 6 months (*p* = 0.212). Overall, recovery trajectories appeared more linear in the BPTB and QT groups, whereas the HT group showed a more variable pattern—with early gains in RF, but delayed improvements in VM and BF—suggesting graft-specific differences in the timing of mechanical recovery [7,45,46,47,48]. These profiles highlight the importance of monitoring both Tc and Dm over time, as graft type not only influences the magnitude of neuromuscular recovery but also its temporal dynamics after ACLR.

These patterns support a targeted rehabilitation strategy based on graft-specific vulnerabilities: quadriceps and hamstring reactivation in QT grafts, posterior chain strengthening in HT grafts, and generalized neuromuscular retraining in BPTB cases. TMG enables the identification of these deficits early and tracks their evolution, offering a valuable guide for individualized post-ACLR recovery plans [6,14,19].

## 5. Limitations

This study presents several limitations that should be acknowledged. First, the relatively small sample size in each graft group (BPTB, HT, QT) limits the statistical power, particularly for subgroup analyses and detection of smaller inter-group differences. While significant trends were observed, the findings should be interpreted with caution and confirmed in larger cohorts.

Second, the study design did not include a healthy control group or the contralateral limb as a reference, which would have provided a clearer benchmark for interpreting the magnitude of Tc and Dm changes. Without this comparative baseline, it is difficult to determine how closely post-operative values approach full recovery. Although a priori power analysis indicated a required sample size of 66 participants, the final cohort consisted of only 31 subjects due to recruitment limitations and clinical constraints. This reduced sample size may limit the statistical power of some comparisons and increase the risk of type II error. Consequently, the results should be interpreted with caution, particularly when comparing outcomes across graft subgroups. Furthermore, the absence of a control group—such as contralateral limbs or healthy participants—limits the internal validity of our findings and precludes causal inference. While this study was intentionally designed as an exploratory observational analysis to assess graft-specific recovery trajectories using TMG, future research should adopt a prospective randomized controlled trial design with appropriate control groups to confirm the effectiveness and clinical utility of graft-specific neuromuscular assessments. The distribution of participants by sex was not balanced across graft groups, with the BPTB group including predominantly males. Given known sex-related differences in neuromuscular function and recovery, this imbalance may have influenced the results independently of graft type. Therefore, caution is warranted when interpreting between-group differences, and future studies should aim to control for sex as a confounding variable.

Third, although TMG offers a non-invasive and reliable assessment of muscle contractility, it evaluates only isolated contractile characteristics and does not fully capture functional performance, such as strength, power, or joint stability. Therefore, the clinical implications of the observed TMG changes remain partially inferential.

Fourth, graft allocation was not randomized, which may have introduced selection bias. Surgeon preference, patient characteristics, or activity level could have influenced graft choice and indirectly affected recovery trajectories.

Lastly, the follow-up period was limited to 9 months. While sufficient to observe early and intermediate recovery patterns, it may not reflect long-term adaptations or late deficits, especially in muscles with delayed remodeling such as the hamstrings in the HT group.

Future studies should include longer-term assessments, larger sample sizes, functional testing, and ideally randomized graft allocation to strengthen the evidence base and expand the clinical relevance of TMG in after ACLR monitoring.

## 6. Clinical Implications

These findings support a graft-specific, muscle-targeted approach to post-ACLR rehabilitation.

First, the persistence of altered Tc and Dm at 9 months in several muscles suggests that time-based protocols may fail to capture incomplete neuromuscular recovery, especially in BPTB and HT cases. TMG provides objective data to detect residual AMI that conventional testing may miss [6,11,12,19,20].

For BPTB grafts, delayed recovery in both quadriceps and BF indicates the need for prolonged, bilateral-chain neuromuscular reactivation. VM and BF should be specifically monitored and addressed, as these muscles showed persistent Tc and Dm impairments [17,39].

In HT grafts, early favorable Tc in BF masked a recovery plateau after 6 months. This supports the integration of long-term, hamstring-focused interventions—eccentric loading, NMES, and explosive coordination drills—extending into the late rehab phase [18,32,38].

QT grafts demonstrated rapid Tc normalization despite early inhibition, especially in RF and VM. This supports early quadriceps reactivation strategies (e.g., NMES, voluntary contraction work) during the first 6–8 weeks post-op [7,20].

TMG also revealed that Tc tends to recover faster than Dm, highlighting the dissociation between neural reactivation and mechanical stiffness [49,50]. Clinically, this suggests adjusting exercise intensity and load progression based not only on strength or ROM but also on TMG markers [51,52].

Finally, graft type should inform both rehab planning and return-to-sport decisions. Rather than applying uniform timelines, clinicians should use TMG to track physiological readiness in specific muscle groups and adapt rehab accordingly. Persistent Tc asymmetry in VM or BF may justify delaying high-risk activities, even when functional tests are satisfactory [6,14,20,53].

## 7. Conclusions

This study provides preliminary evidence that contractile properties of the quadriceps and hamstrings evolve significantly between 3 and 9 months post-ACL reconstruction, and that different graft types may influence the neuromuscular recovery trajectory. However, due to the study’s limitations—including a small sample size, unbalanced sex distribution across groups, and the absence of randomization or control group—these findings should be interpreted with caution.

While the results highlight trends that may inform future rehabilitation strategies and graft selection, they cannot support definitive clinical recommendations at this stage. Larger, controlled, and sex-balanced studies are needed to confirm these observations and clarify the role of graft type in neuromuscular recovery after ACLR.

## Figures and Tables

**Table 1:** Baseline characteristics by graft type.

	BPTB GROUP n = 8	HT GROUP n = 12	QT GROUP n = 11	TOTAL n = 31
**Gender**				
Male	8	7	0	15
Female	0	5	11	16
**Age (years)**				
Mean	22.38	21.75	21.27	21.74
SD	1.51	1.66	1.79	1.67
**Weight (kg)**				
Mean	69.88	67.83	59.91	65.55
SD	8.39	12.97	8.46	10.98
**Height (cm)**				
Mean	173.75	175.75	166.82	172.06
SD	5.97	12.17	8.22	10.07
**BMI**				
Mean	23.08	21.78	21.43	21.99
SD	1.44	1.85	1.55	1.73

Note: BPTB; bone–patellar tendon–bone, HT; hamstring tendon, QT; quadriceps tendon, M; male, F; female, kg; kilograms, m; meters, BMI; body mass index (kg/m^2^), SD; standard deviation.

**Table 2:** TMG follow-up parameters. BPTB, bone–patellar tendon–bone; HT, hamstrings tendon; QT, quadriceps tendon; RF, rectus femoris; VM, vastus medialis; VL, vastus lateralis; BF, biceps femoris; TC, contraction time; DM, maximal displacement; SD, standard deviation.

				BPTB Group *n* = 8	HT Group *n* = 12	QT Group *n* = 11	Kruskal–Wallis H Test *p* Value	Dunn’s Post Hoc Test (Bonferroni Correction)		
								BPTP-HT	BPTP-QT	HT-QT
**RF**	TC (ms) mean ± SD	TC 3 months		31.84 ± 0.80	26.37 ± 1.93	30.59 ± 1.33	*<0.001*	<0.001	*0.447*	*0.004*
		TC 6 months		22.14 ± 2.45	21.20 ± 2.08	22.83 ± 1.43	*0.052*			
		TC 9 months		21.16 ± 0.65	16.73 ± 1.48	15.86 ± 0.70	*<0.001*	*0.004*	<0.001	*0.725*
		**Friedman Test (*p* value)**		*0.002*	*<0.001*	*<0.001*				
		**Conover’s post hoc test**	**3–6 months**	*<0.001*	*<0.001*	*<0.001*				
			**6–9 months**	*0.146*	*<0.001*	*<0.001*				
			**3–9 months**	*<0.001*	*<0.001*	*<0.001*				
	DM (mm) mean ± SD	DM 3 months		5.12 ± 0.93	6.01 ± 0.53	5.79 ± 0.73	*0.033*	*0.027*	*0.436*	*0.650*
		DM 6 months		6.17 ± 0.86	6.39 ± 0.04	6.65 ± 0.62	*0.068*			
		DM 9 months		7.01 ± 0.44	7.52 ± 0.52	7.43 ± 0.74	*0.065*			
		**Friedman Test (*p* value)**		*0.005*	*<0.001*	*0.003*				
		**Conover’s post hoc test**	**3–6 months**	*0.182*	*0.551*	*0.084*				
			**6–9 months**	*0.017*	*<0.001*	*0.084*				
			**3–9 months**	*<0.001*	*<0.001*	*<0.001*				
**VM**	TC (ms) mean ± SD	TC 3 months		31.33 ± 0.56	25.76 ± 3.32	30.84 ± 0.85	*<0.001*	*<0.001*	*0.849*	*0.001*
		TC 6 months		22.59 ± 2.79	20.18 ± 1.90	24.02 ± 2.99	*<0.001*	*0.071*	*0.881*	*<0.001*
		TC 9 months		21.28 ± 1.05	18.37 ± 5.82	18.45 ± 2.92	*0.017*	*0.013*	*0.182*	*0.923*
		**Friedman Test (*p* value)**		*0.002*	*<0.001*	*<0.001*				
		**Conover’s post hoc test**	**3–6 months**	*<0.001*	*<0.001*	*<0.001*				
			**6–9 months**	*1.000*	*0.304*	*0.004*				
			**3–9 months**	*<0.001*	*<0.001*	*<0.001*				
	DM (mm) mean ± SD	DM 3 months		5.16 ± 0.78	5.73 ± 0.53	5.61 ± 0.62	*0.025*	*0.020*	*0.278*	*0.823*
		DM 6 months		5.90 ± 0.59	6.66 ± 0.51	6.61 ± 0.59	*0.018*	*0.015*	*0.136*	*1.000*
		DM 9 months		6.88 ± 0.85	7.15 ± 0.47	7.30 ± 0.69	*0.450*			
		**Friedman Test (*p* value)**		*0.002*	*<0.001*	*0.002*				
		**Conover’s post hoc test**	**3–6 months**	*0.013*	*<0.001*	*0.030*				
			**6–9 months**	*0.013*	*0.049*	*0.084*				
			**3–9 months**	*<0.001*	*<0.001*	*<0.001*				
**VL**	TC (ms) mean ± SD	TC 3 months		29.18 ± 3.67	28.28 ± 2.35	30.48 ± 0.31	*0.011*	*0.033*	*1.000*	*0.031*
		TC 6 months		22.59 ± 2.79	20.18 ± 1.90	24.02 ± 2.99	*0.008*	*0.142*	*1.000*	*0.008*
		TC 9 months		19.24 ± 3.27	17.12 ± 3.05	17.48 ± 1.79	*0.160*			
		**Friedman Test (*p* value)**		*0.001*	*<0.001*	*<0.001*				
		**Conover’s post hoc test**	**3–6 months**	*<0.001*	*<0.001*	*<0.001*				
			**6–9 months**	*0.019*	*0.037*	*<0.001*				
			**3–9 months**	*<0.001*	*<0.001*	*<0.001*				
	DM (mm) mean ± SD	DM 3 months		5.07 ± 0.81	6.00 ± 1.30	5.79 ± 0.55	*0.076*			
		DM 6 months		6.01 ± 0.47	6.73 ± 0.61	6.44 ± 0.58	*0.034*	*0.028*	*0.458*	*0.637*
		DM 9 months		7.03 ± 0.12	7.32 ± 0.28	7.44 ± 0.63	*0.007*	*0.006*	*0.059*	*1.000*
		**Friedman Test (*p* value)**		*<0.001*	*0.005*	*<0.001*				
		**Conover’s post hoc test**	**3–6 months**	*0.002*	*0.093*	*0.382*				
			**6–9 months**	*<0.001*	*0.212*	*0.001*				
			**3–9 months**	*<0.001*	*0.001*	*<0.001*				
**BF**	TC (ms) mean ± SD	TC 3 months		31.25 ± 0.73	24.34 ± 3.03	30.09 ± 3.13	*<0.001*	*0.001*	*1.000*	*0.009*
		TC 6 months		28.02 ± 4.41	20.17 ± 2.15	21.45 ± 1.22	*<0.001*	*<0.001*	*0.108*	*0.245*
		TC 9 months		18.04 ± 3.72	20.08 ± 4.62	18.37 ± 3.22	*0.586*			
		**Friedman Test (*p* value)**		*0.005*	*0.002*	*<0.001*				
		**Conover’s post hoc test**	**3–6 months**	*0.380*	*<0.001*	*<0.001*				
			**6–9 months**	*0.012*	*1.000*	*0.101*				
			**3–9 months**	*<0.001*	*0.002*	*<0.001*				
	DM (mm) mean ± SD	DM 3 months		5.12 ± 0.79	5.37 ± 0.19	5.86 ± 0.81	*0.031*	*0.230*	*0.027*	*0.990*
		DM 6 months		6.11 ± 0.72	6.68 ± 0.42	6.59 ± 0.42	*0.172*			
		DM 9 months		6.92 ± 0.29	7.24 ± 0.53	7.42 ± 0.62	*0.050*			
		**Friedman Test (*p* value)**		*0.002*	*<0.001*	*0.002*				
		**Conover’s post hoc test**	**3–6 months**	*0.013*	*<0.001*	*0.164*				
			**6–9 months**	*0.013*	*0.238*	*0.022*				
			**3–9 months**	*<0.001*	*<0.001*	*<0.001*				

## Data Availability

The data supporting the findings of this study are available upon reasonable request from the corresponding author, Dr Florian Forelli (florian.forelli@he-arc.ch). Data are not publicly available due to confidentiality agreements and the presence of sensitive medical information.
